# Body-states: interpersonal and relational perspectives on the treatment of eating disorders – edited by Jean Petrucelli

**DOI:** 10.1186/s40337-016-0089-z

**Published:** 2016-02-11

**Authors:** Beth Shelton

**Affiliations:** Victorian Centre of Excellence in Eating Disorders, Parkville, VIC Australia

In this substantial anthology of essays, editor Jean Petrucelli draws together the contributions of many clinicians and researchers working from an interpersonal perspective with eating disorders. The book is Petrucelli’s compendium and arises as a result of her own long-term work in the field and that of many colleagues. It is structured into five parts by theme, each part comprised of between two and six essays.

- Part 1: Finding Beauty in the Beast: Interpersonal Perspectives and Treatment of Eating Disorders
- Part 2: The Mindbody and the Bodymind: Reflections Inside and Outside Reality and Subjectivity
- Part 3: Treating the Family, the Young, the Hormonal, and the Religious: Developmental, Familial, and Cultural Contexts
- Part 4: Appetite Regulation in an Interpersonal and Cultural Context
- Part 5: Beyond the Interpersonal: Clinical and Assessment Tools Across Modalities

Petrucelli tells the reader in her introduction that the overarching theme of the book is the role of body states and their relational meanings in the understanding and treatment of eating disorders. This theme is indeed a compelling conceptual and clinical thread across the essays. The account given of body-based approaches to eating and body image problems is perhaps the most groundbreaking aspect of the book. The protocol for the BODI (Body Observational Diagnostic Interview) is one of the most useful.

The book provides a conceptually rich and clinically useful account of a groundswell of interest and clinical expertise in an interpersonal and integrative approach to eating disorders. This volume is not a treatment manual but it is rich in clinical wisdom and example. It is not meant to be read in a sitting but to be a source of enrichment to be savoured and dipped into over time.

A broad menu of topics includes the interface of self and affect regulation with neurobiology and attachment, bodies in interaction, body image and the reflexive mind, obesity and binge eating, sustainability in recovery and clinical applications and techniques. Case studies of treatment are provided, often characterized by detailed behavioural activation in the context of a therapeutic relationship allowing for intrapersonal and interpersonal depth. There is much to learn and reflect upon in this book about the predicament and potential of embodied life and the process of change for those experiencing eating disorders.

As a clinician, researcher, consumer, carer or general reader interested in eating disorders and embodied life, what might this book offer? This is a tricky question to answer as the complexity and richness of the information and insight in the essays will provide every reader with a different journey. Speaking generally then, the book may offer an increased appreciation of the unique individual experiencing an eating disorder. It may help with reflection about the way each person’s eating disorder may be intrinsically linked to unspoken interpersonal bargains made in response to developmental experiences. It may provide an increased understanding of both positive and problematic experiences of embodiment. Examples of the former include being alive in and connected to one’s body, with a stable body image flexibly integrative of fluctuating body states. Problematic experiences of embodiment include mind-body splits and the simultaneous loathing, and stifling identity conflation with body that many eating disorder patients experience.

The book has been written in a style that is comprehensible for a reader not from the analytic tradition. Attention has been given to user-friendly writing and to embedding explanation of key concepts within articles. This is a generous, integrative, conceptually rich book. It gives a contemporary view of interpersonal approaches to eating disorders. If you would like an invitation to reflection over time to deepen and inform your practice and understanding, this book would be for you.

